# Rectal Microbiomes and Serum Metabolomics Reveal Changes in Serum Antioxidant Status and Immune Responses of Dezhou Donkeys in Late Gestation to Parturition

**DOI:** 10.3390/antiox14101253

**Published:** 2025-10-18

**Authors:** Fang Hui, Yanli Zhao, Zaccheaus Pazamilala Akonyani, Yongmei Guo, Xiaoyu Guo, Qingyue Zhang, Fanzhu Meng, Li Li, Binlin Shi, Sumei Yan

**Affiliations:** Key Laboratory of Animal Nutrition and Feed Science of Inner Mongolia Autonomous Region, College of Animal Science, Inner Mongolia Agricultural University, Hohhot 010018, China; hf2021@emails.imau.edu.cn (F.H.); zakonyani@emails.imau.edu.cn (Z.P.A.); ymguo2020@imau.edu.cn (Y.G.); gxy2024@imau.edu.cn (X.G.); alicezqy@emails.imau.edu.cn (Q.Z.); fanzhumeng@emails.imau.edu.cn (F.M.); lily972021@emails.imau.edu.cn (L.L.); shibl@imau.edu.cn (B.S.)

**Keywords:** late gestation, rectal microbiota, serum metabolome, Dezhou donkeys, antioxidant capacity, anti-inflammatory response

## Abstract

Parturition is a critical event in the reproductive cycle of dairy animals, accompanied by multiple physiological changes in sex hormones, metabolism, antioxidant capacity, and immune function. However, the changes in the rectal microbiota and metabolic products of Jennies from late gestation to parturition affect serum antioxidant capacity and anti-inflammatory responses, but it is still unclear. The present study aimed to investigate the serum antioxidant capacity and anti-inflammatory responses of Dezhou donkeys from late gestation to parturition by analyzing rectal microbiomes and serum metabolomics. Nine pregnant multiparous Dezhou Jennies, aged 6.0 ± 0.1 years, with a body weight of 292 ± 33 kg, an average parity number of 2.7 ± 0.1, and similar expected dates of confinement (35 ± 4 days), were selected for this study. The study investigates the changes in antioxidant capacity and inflammatory responses, as well as the alterations in rectal microbiota structure and serum metabolites, in Jennies at 35 days prepartum (B1), 7 days prepartum (B2), and at 0 h postpartum (B3). The results showed that from groups B1 to B2, serum activity of GSH-Px, IL-10, and GLU concentrations were decreased significantly. In contrast, the concentrations of MDA, IgG, LF, IL-1β, IL-2, IL-6, TNF-α, and ROS increased significantly. From groups B2 to B3, serum activities of GSH-Px, CAT, SOD, and T-AOC, as well as the concentrations of MDA, IgG, IL-2, AST, ALP, and BHBA, were significantly increased, whereas the concentrations of IL-4, IL-10, and CRE decreased considerably. Therefore, from 35 days prepartum to parturition, Jennies experienced a gradually intensifying oxidative stress and inflammatory states, with the inflammatory response being the most severe at parturition, and with enhanced antioxidant capacity corresponding to increased oxidative damage. Microbiome analysis revealed that the group B1 significantly increased the relative abundance of *Prevotella* and *Fibrobacteres*. Group B2 significantly increased the relative abundance of *Prevotellaceae_UCG-001*, *Streptococcus*, and *Acetitomaculum*. Group B3 showed a significant upregulation of the relative abundance of *Norank_f__F082*, *Lachnospiraceae_UCG-009*, and *Prevotellaceae_UCG-004*. At the same time, metabolomics analysis revealed that, compared with group B1, group B3 may alleviate inflammation and enhance the body’s antioxidant function by upregulating the tryptophan and arginine metabolic pathways and enriching the differential metabolites (L-tryptophan, L-kynurenine, 3-Indoleacetonitrile, N-acetylglutamic acid). Concurrently, the elevation of these differential metabolites may be associated with the relative abundance of the beneficial bacterium *Lachnospiraceae_UCG-009*. However, the increase in LysoPC, a fatty acid oxidation product in glycerophospholipid metabolism, as well as the correlation between the sucrose content in the galactose metabolic pathway and the abundance of *Paracoccus*, indicates the reason why the Jennies are in a state of oxidative stress. Furthermore, group B1 may enhance the serum anti-inflammatory response in Jennies during late gestation by increasing the levels of estrogen in the steroid hormone biosynthesis metabolic pathway. These results could provide useful information for improving the health levels at the specific physiological stages and processes in Dezhou donkeys.

## 1. Introduction

Donkey *(Equus asinus*) milk is a functional food that can serve as a substitute for breast milk in newborns due to its chemical composition, which is similar to that of human milk, and its low allergenicity. It also helps prevent hypercholesterolemia and atherosclerosis [1]. However, the intensive farming systems for donkeys have yet to be as well-established as those for other livestock breeds. Breeding standards and corresponding supporting management systems are not yet fully established. The period from late gestation to lactation is one of the most important physiological stages in the life course of female mammals. During this time females experience the processes of gestation, parturition, and lactation, along with significant changes in physiology, endocrinology, and the immune system. In ruminants (dairy cows), the periparturient period is often in a state of stress due to factors such as parturition, lactation, dietary structure, and environmental changes, leading to increased immunosuppression and disease susceptibility, which not only reduces milk production and affects milk quality but also causes significant economic losses to farmers [2,3]. In sows, particularly around the time of farrowing, the oxidative/antioxidant balance is disrupted, leading to oxidative stress, which not only affects milk quality but may also hurt piglet growth [4]. In addition, reports have also indicated that from 7 days prepartum to parturition, the concentrations of non-esterified fatty acids (NEFA), β-hydroxybutyric acid (BHBA), and tumor necrosis factor-α (TNF-α) in cow plasma significantly increased, while the concentrations of immunoglobulin G (IgG) and interleukin (IL)-2 in plasma significantly decreased [5]. Studies have shown that this process places Jennies at greater risk of developing metabolic diseases [6]. Therefore, understanding the changing patterns of antioxidant capacity and anti-inflammatory responses from late gestation to parturition is critical for improving the performance of Jennies.

In recent years, an increasing number of studies have shown that the role of microbiome and metabolomics in animal health and performance cannot be ignored. Intestinal flora is not only involved in host metabolic processes but also influences animal health by modulating immune responses and antioxidant status [7,8]. Significant changes in the microbiota of periparturient dams have been observed in studies involving sows and humans [9,10], revealing a relationship between perinatal gut flora and maternal oxidative stress as well as inflammatory status [11,12]. Li et al. [13] reported that there is a complex relationship between the microbiota and gene expression, especially concerning the immune system. Guo et al. [14] found that the rectal flora structure of female donkeys and antioxidant and inflammatory status during late gestation were influenced by the dietary energy levels. These findings suggest that changes in microbiota and their associated metabolic alterations may serve as potential mechanisms for inducing antioxidant and anti-inflammatory effects and their corresponding phenotypes.

It has also been reported that the gut microbiota influences multiple metabolic pathways [15]. Li et al. [16] also found that the serum metabolite choline was negatively correlated with the abundance of *Lactobacillus*, *Ruminalococcus*, and unclassified genera of *Prevotellaceae* in the rectal flora. These findings suggest that changes in the gut microbiota and its metabolic alterations may be a potential mechanism for regulating the microbiome–metabolome axis of immune and antioxidant properties, with the essential bacteria and key metabolite-driven rectal microbiome playing an important role. In the present study, it was hypothesized that Jennies exhibit significant changes in serum antioxidant capacity and immune function during late gestation and parturition, and that the mechanisms of these changes may be related to alterations in bacteria and metabolites in the Jennies. Therefore, this study aims to validate the significant changes in serum antioxidant capacity and immune function from late gestation to parturition and to modulate the role of the “microbiome–metabolome” axis. To this end, we will integrate various omics techniques, including rectal microbiomics and serum metabolomics, to provide a scientific basis for improving the gastrointestinal microecology, immune function, and performance of Jennies.

## 2. Materials and Methods

### 2.1. Ethical Approval Declarations

All animal procedures were performed under the special committee on scientific research and technology ethics of Inner Mongolia Agricultural University, with approval code [2019]035, dated 26 December 2019. All procedures were conducted in accordance with relevant ethical guidelines to ensure animal welfare.

### 2.2. Experimental Design and Treatments

Nine pregnant multiparous Dezhou Jennies, aged 6.0 ± 0.1 years, with a body weight of 292 ± 33 kg, an average parity number of 2.7 ± 0.1, and similar expected dates of confinement (35 ± 4 days), were selected for this study. The study investigates the changes in antioxidant capacity and inflammatory responses, as well as the alterations in rectal microbiota structure and serum metabolites, in Jennies at 35 days prepartum (B1), 7 days prepartum (B2), and at 0 h postpartum (B3). The ingredients and nutrient compositions of the diets are shown in Table 1. The experiment lasted 44 days (comprising a 14-day adaptation period and a 30-day experimental phase). The Jennies were kept in individual pens and fed twice daily at 07:30 and 14:00, respectively. The concentrate-to-forage ratio of the ration was kept constant at 30 to 70. The Jennies had free access to the diets and water.

### 2.3. Sample Collection

Blood samples were collected from the jugular vein of each jenny at 35 days (Group B1) and 7 days prepartum (Group B2), as well as at 0 h (Group B3) postpartum. A 10 mL non-anticoagulant vacuum tube (Corning™, Costar^®^, Corning Inc., New York, NY, USA) was used for blood collection. After collection, the tube was placed on ice to allow stratification. Subsequently, serum was separated by centrifugation at 2054.3× *g* for 15 min at 4 °C, and the samples were stored at −20 °C for subsequent antioxidant, immunologic, and biochemical measurements.

Serum Antioxidant Indicators. The following indicators were measured: glutathione peroxidase (GSH-Px, A005–1-2), superoxide dismutase (SOD, A005–1-2), catalase (CAT, A005–1-2), total antioxidant capacity (T-AOC, A015–1-2), malondialdehyde (MDA, A003–1-2) content, and reactive oxygen species (ROS) concentration. According to the manufacturer’s instructions, these analyses were conducted using commercial detection kits from Nanjing Jingke Bioengineering Institute, Nanjing, China.

Serum Immune Indicators. The following immune indicators were measured: immunoglobulin G (IgG), Lactoferrin (LF), interleukin (IL)-1β, IL-2, IL-4, IL-6, IL-10, and tumor necrosis factor-α (TNF-α). Measurements were performed using enzyme-linked immunosorbent assay (ELISA) kits from the Beijing SINO-UK Institute of Biological Technology (Beijing, China), as well as commercial detection kits from Nanjing Jingke Bioengineering Institute, which were operated following the manufacturer’s instructions.

Serum Biochemicals. The following biochemical markers were analyzed: alanine aminotransferase (ALT), aspartate aminotransferase (AST), alkaline phosphatase (ALP), total protein (TP), albumin (ALB), urea (UREA), creatinine (CRE), glucose (GLU), cholesterol (CHO), and β-hydroxybutyric acid (BHBA). The analyses were performed using kits supplied by Lepu Pharmaceutical (Beijing, China) Co., Ltd., on a Hitachi 7020 automatic biochemical analyzer (Hitachi, Tokyo, Japan), and operated following the manufacturer’s instructions.

### 2.4. Rectal Microbiota

Rectal feces were collected from all animals at the 3 different times points. These samples were stored in DNase- and RNase-free tubes (Shanghai Precision Chemical Technology Co., Ltd., Shanghai, China) and immediately frozen in liquid nitrogen (−196 °C) for 16S microbiome analysis.

Rectal Microbiome Analysis. ① DNA extraction and PCR amplification: Total DNA from rectal contents was extracted from fecal samples according to the E.Z.N.A. Soil DNA Kit (Omega, Bio-Tek, Norcross, GA, USA) instruction manual. The quality of the DNA extracted was detected by 1% agarose gel electrophoresis, and DNA concentration and purity were determined using a NanoDrop 2000 (Thermo Scientific, Wilmington, DE, USA). Sequencing primers 338F (5′-ACTCCTACGGGGAGGCAG-CAG-3′) and 806R (5′-GGACTACHVGGGTWTCTAAT-3′) were selected for the 16S rRNA gene amplification, targeting the V3 to V4 variable regions by PCR. The PCR procedure is as follows: initial denaturation at 95 °C for 3 min, followed by 27 cycles of denaturing at 95 °C for 30 s, annealing at 55 °C for 30 s and extension at 72 °C for 45 s, and single extension at 72 °C for 10 min, and end at 4 °C (PCR instrument: ABI GeneAmp^®^ 9700, ABI, Foster City, CA, USA). In the PCR amplification step, sterile water was used as the negative control. The absence of a CP band in the amplification report indicates that there was no environmental contamination during the process. ② Sequencing on the Illumina MiSeq platform: the PCR products of the same sample were mixed, and the bands were separated by 2% agarose gel electrophoresis, purified using the AxyPrep DNA Gel Extraction Kit (Axygen Biosciences, Union City, CA, USA), and sequenced by a fluorometer (Quantus™ Fluorometer, Promega, Madison, WI, USA). MiSeq library preparation was performed using the NEXTFLEX Rapid DNA-Seq Kit (Bioo Scientific, Austin, TX, USA). Following successful Qubit and Q-PCR quantification validation, the prepared libraries underwent sequencing on the MiSeq PE300 platform (Shanghai Meiji Biotechnology Co., Ltd. Shanghai, China). ③ Use fastp software (version 0.20.0) to perform quality control on raw sequences, removing any sequences shorter than 50 bp after quality control. Subsequently, employ FLASH software (version 1.2.7) for assembly: align the sequences at both ends based on overlapping bases, requiring an overlap greater than 10 bp, and discard sequences that cannot be assembled. After OTU classification (with a similarity level of 97%) was performed using Uparse (version 7.0.1090, http://drive5.com/uparse/ accessed on 13 October 2024). Alpha-diversity analysis was carried out using Mothur (https://www.mothur.org/wiki/Download_mothur, accessed on 13 October 2024), Venn diagram analysis (in R, version 3.3.1, accessed on 13 October 2024), and β-diversity analysis was performed with QIIME (http://qiime.org/install/index.html, accessed on 13 October 2024). Also, community composition analysis and tests of significant differences between groups (at the phylum and genus level), LEfSe analysis, calculation of correlation coefficients between environmental factors and remarkable genera (Spearman rank correlation coefficients and representation of the corresponding numerical matrices by heat maps. The colors indicate the magnitude of the data values in the matrix or table. Both the calculations and visualization can be performed using the vegan package in R (version 3.3.1).

### 2.5. Serum Metabolome

Metabolic samples were collected from groups B1 and B3. Blood samples were collected in 2 mL cryotubes (Corning Inc., Costar, New York, NY, USA), snap-frozen, transferred to an ultra-low-temperature freezer, and stored at −80 °C for metabolomics analysis. Untargeted Liquid Chromatography-MS Metabolomic analysis. Metabolomic analysis of the plasma samples was performed using a liquid chromatography (LC)-mass spectrometry (MS) (Thermo Scientific, Waltham, MA, USA, Vanquish UHPLC System-Q Exactive HF-X) platform. The samples were tested according to the methodology of Li et al. [16] and sequenced in accordance with the standard protocol provided by Shanghai Precision Chemical Technology Co., Ltd. (Shanghai, China). It is worth noting that equal quantities of all sample metabolites are pooled to prepare quality control (QC) samples. During instrumental analysis, one QC sample was inserted every three samples to assess the repeatability of the entire analytical process.

Metabolite identification and screening were conducted by matching MS and MS/MS information with reliable biochemical databases, such as the Human Metabolome Database (http://www.hmdb.ca, accessed on 21 October 2024) and the METLIN database (https://metlin.scripps.edu, accessed on 21 October 2024). Metabolite pathway analyses were conducted on the Kyoto Encyclopedia of Genes and Genomes (KEGG) database (http://www.genome.jp/kegg/, accessed on 21 October 2024). In addition, the Majorbio Cloud Platform (https://cloud.majorbio.com, accessed on 21 October 2024) was applied as the data analysis tool.

### 2.6. Statistical Analysis

The data for blood antioxidant indices, immune indices, and biochemical indices, as well as bacterial diversity indices (Coverage, Chao, ACE, Shannon, and Simpson) were analyzed using the analysis of variance (ANOVA) in SAS (SAS Software, Version 9.1; SAS Institute, Cary, NC, USA), followed by Duncan’s multiple range test set at the 0.05 level of significance (*p* ≤ 0.05). Kruskal–Wallis H test sums were utilized to analyze differences among phyla, families, and genera of the rectal bacteria. Additionally, the rectal microbial abundance of Jennies at the genus level was assessed using Linear Discriminant Analysis Effect Size (LEfSe), with a threshold LDA score exceeding 3 for significance. Differentially expressed metabolites were identified based on a fold change (FC) > 1 and *p* < 0.05. Significant differences were defined as *p* < 0.05, while *p*-values in the 0.05 ≤ *p* < 0.10 range were regarded as indicating a trend toward significance. Spearman correlation was used to correlate antioxidant indices, immune indices, biochemical indices, and differential metabolites with the result of LEfSe bacterial genera using R (heatmap package, version 3.3.1).

## 3. Results

### 3.1. Serum Antioxidative Status, Inflammatory Cytokines, Immunoglobulin, and Biochemical Parameters

**Figure 1:**
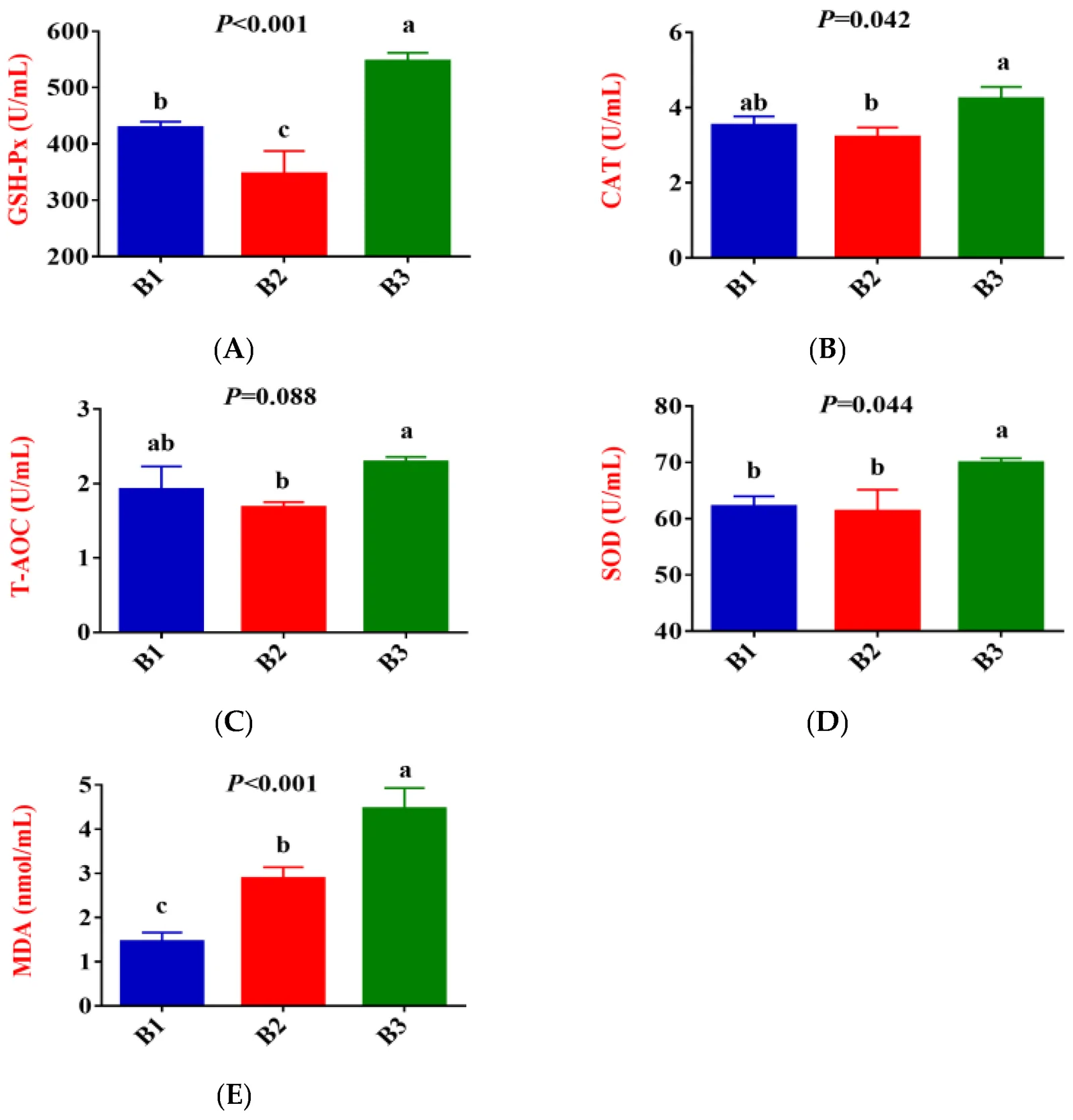
Comparison of serum antioxidant indices between the 3 groups. (B1 = 35 days prepartum, B2 = 7 days prepartum, B3 = 0 h postpartum). (**A**) GSH-Px = Glutathione peroxidase; (**B**) CAT = Catalase; (**C**) T-AOC = Total antioxidant capacity; (**D**) SOD = Superoxide dismutase; (**E**) MDA = Malondialdehyde; a, b, and c mean that the 3 groups that do not have a common marked letter differ significantly (*p* < 0.05).

Figure 1 shows that, compared to group B1, the activities of GSH-Px in the serum of group B2 significantly decreased, while the concentration of MDA exhibited an upward trend (*p* < 0.05). Compared to group B2, the activities of GSH-Px, CAT, SOD and T-AOC, and MDA contents in the serum of group B3 significantly increased.

**Figure 2:**
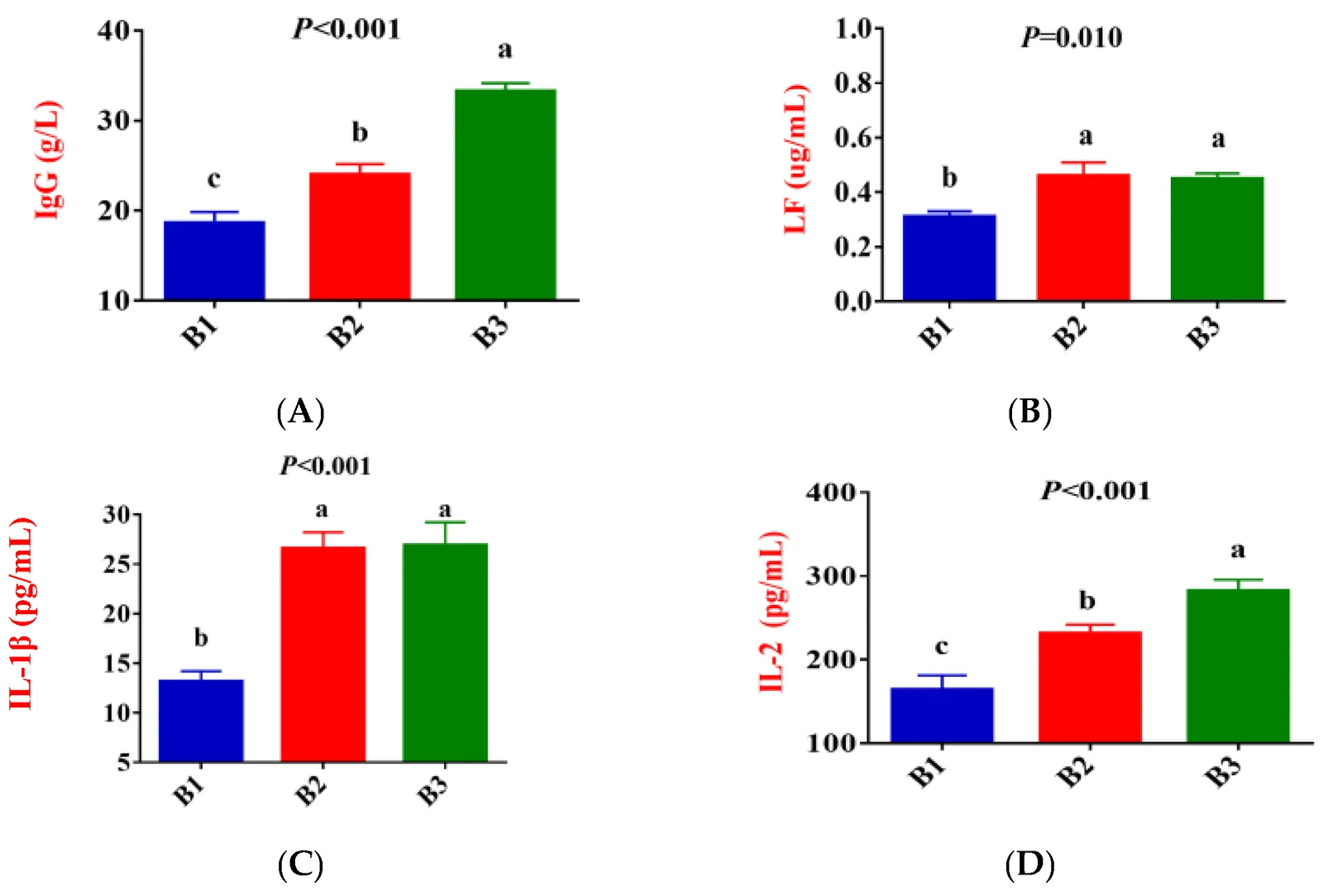

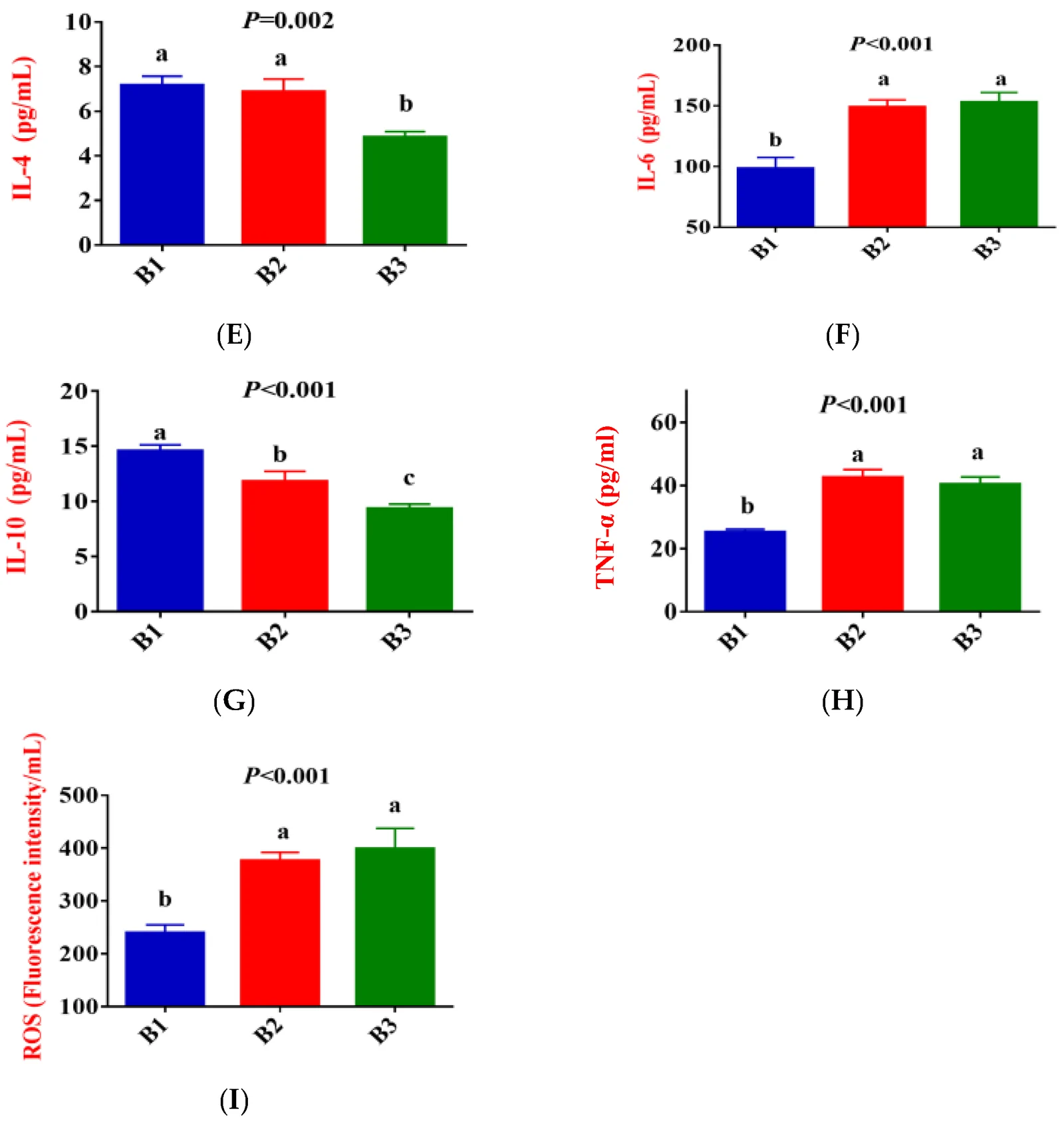
Comparison of serum immune indicators between the 3 groups. (B1 = 35 days prepartum, B2 = 7 days prepartum, B3 = 0 h postpartum). (**A**) IgG = Immunoglobulin G; (**B**) LF = Lactoferrin; (**C**) IL-1β = Interleukin-1β; (**D**) IL-2 = Interleukin-2; (**E**) IL-4 = Interleukin-4; (**F**) IL-6 = Interleukin-6; (**G**) IL-10 = Interleukin-10; (**H**) TNF-α = Tumor necrosis factor-α; (**I**) ROS = Reactive oxygen species; a, b, and c mean that the 3 groups that do not have a common marked letter differ significantly (*p* < 0.05).

Figure 2 shows that, compared to group B1, the concentration of IgG, LF, IL-1β, IL-2, IL-6, TNF-α, and ROS in the serum of group B2 significantly increased, while the concentration of IL-10 exhibited a downward trend (*p* < 0.05). Compared to group B2, the concentration of IgG and IL-2 in the serum of group B3 significantly increased, while the concentration of IL-4 and IL-10 exhibited a downward trend (*p* < 0.05). There was no difference in LF, IL-1β, IL-6, TNF-α, and ROS concentrations between the B2 and B3 groups.

Table 2 shows that, compared to group B1, the concentrations of GLU in the serum of group B2 significantly decreased (*p* < 0.05). There were no significant differences in ALT, AST, ALP, TP, ALB, UREA, CRE, and BHBA concentrations between groups B1 and B2.

Compared to group B2, the concentrations of AST, ALP, and BHBA in the serum of group B3 significantly increased, while the concentrations of CRE significantly decreased. There was no difference in GLU concentrations between groups B2 and B3.

### 3.2. Rectal Bacterial Community Richness, Diversity, and Composition

**Figure 3:**
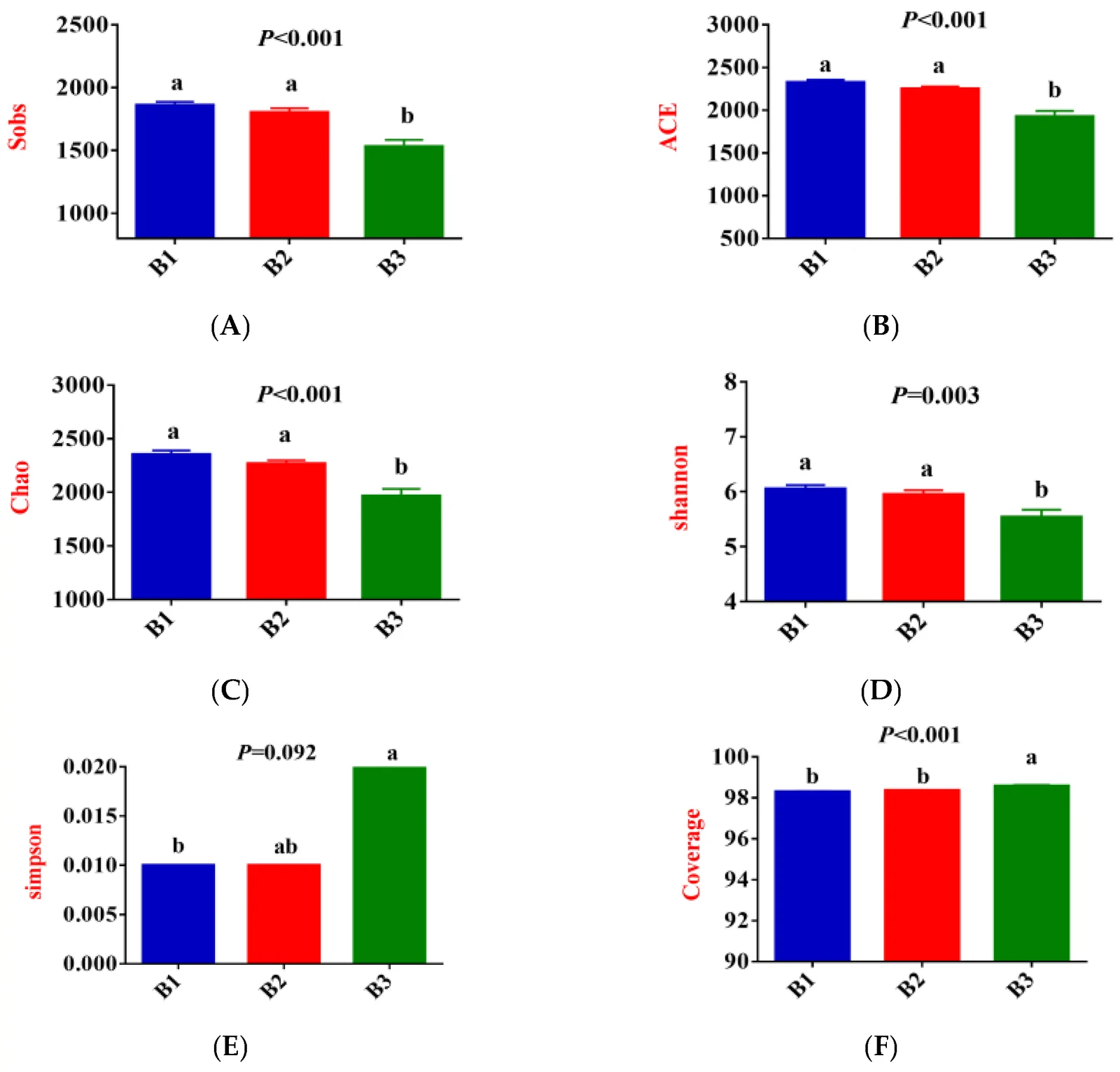

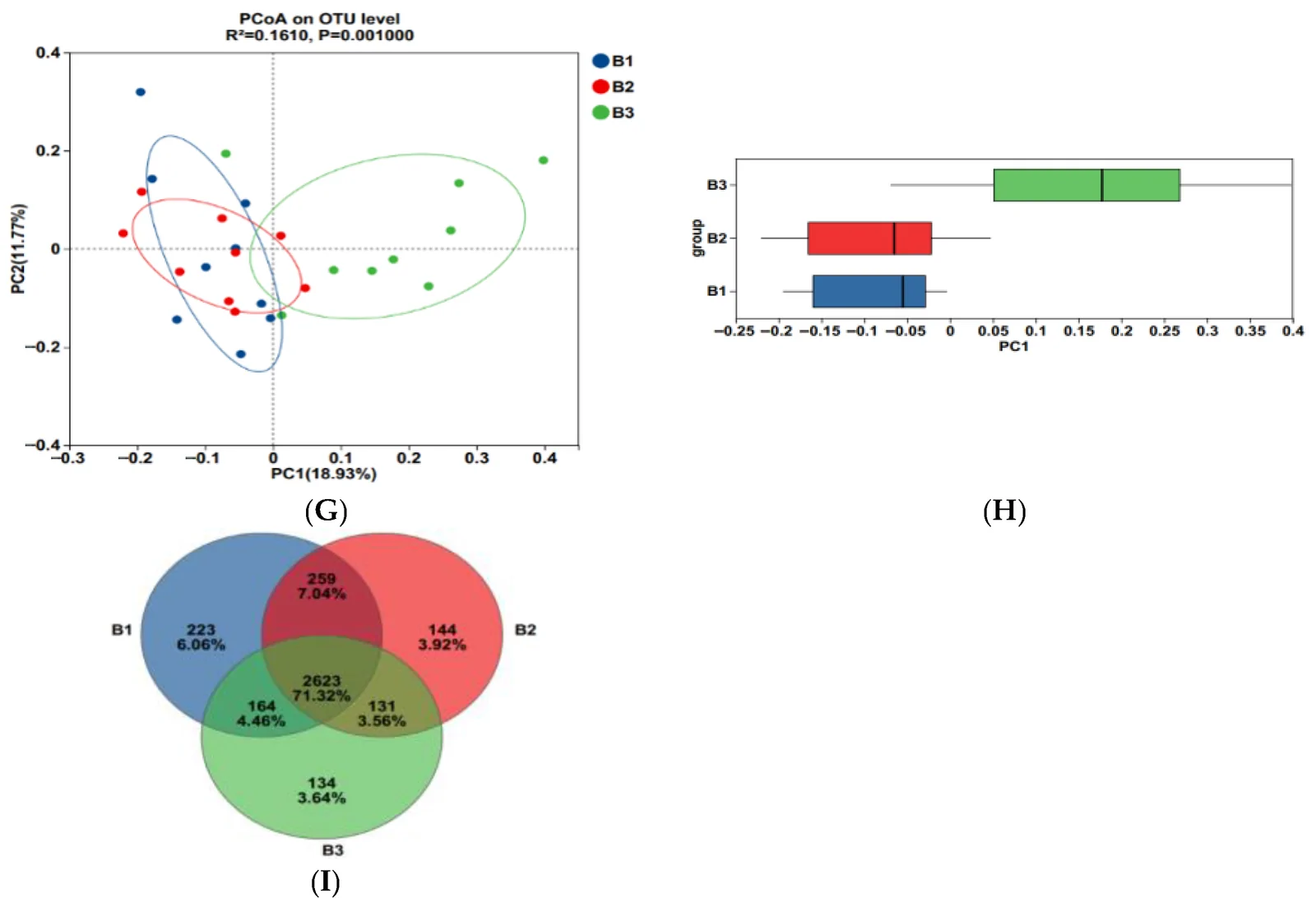
The alpha-diversity index of rectal microorganisms (**A**–**F**). Principal Coordinate Analysis (PCoA) (**G**,**H**); Venn diagram of the OTUs in the rectal microbiota (**I**). B1 = 35 days prepartum; B2 = 7 days prepartum; B3 = 0 h postpartum. a and b mean that the 3 groups that do not have a common marked letter differ significantly (*p* < 0.05).

As shown in Figure 3A–F, Sobs, ACE, Chao, and Shannon indices were significantly greater in groups B1 and B2 than those recorded in group B3, while the coverage indices show the opposite trend (*p* < 0.05); Simpson index for group B3 was significantly higher than that for group B1 (*p* < 0.05), while group B2 tended to fall between the two groups. Furthermore, the community coverage reached 98% for all 3 test groups, indicating that rectal microorganisms’ species and structural diversity could be accurately assessed.

Principal coordinate analysis (PCoA) based on the Bry-Curtis distance matrix revealed that the groups B3 rectal microbial samples had more variation than the groups B1 and B2, with PC1 and PC2 accounting for 18.93% and 11.77% of the total variation, respectively (Figure 3G,H), indicating that there were differences in rectal microbial species.

In this experiment, non-repeated sequences were clustered using OTUs based on 97% similarity, which produced 3125 OTUs. Additionally, 223, 144, and 134 unique OTUs were detected in groups B1, B2, and B3, respectively, making a total of 2623 OTUs among the 3 experimental groups (Figure 3I).

### 3.3. Significantly Different Rectal Bacteria Among Groups B1, B2, and B3

**Figure 4:**
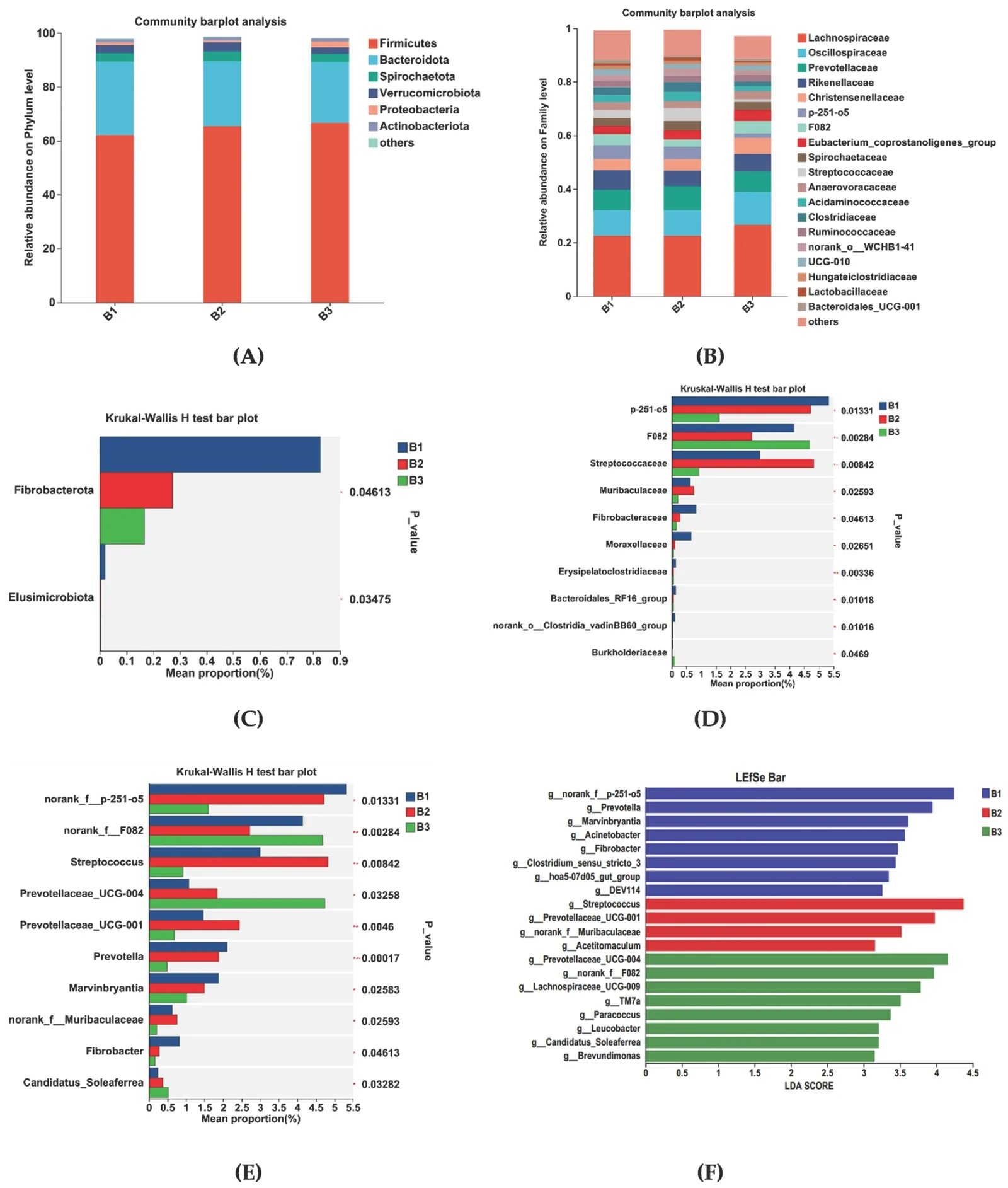
Differential bacterial compositional functions among B1, B2, and B3 Jennies. The relative abundances of rectal bacteria are at the (**A**) phylum and (**B**) family levels. Differential bacterial compositions at the (**C**) phylum, (**D**) family, and (**E**) genus levels using 16S rRNA sequence data based on the Kruskal–Wallis H test. LEfSe analysis of rectal microbiota among 3 treatments. (**F**) Linear discriminant analysis (LDA) value distributed histogram, and the score ≥ 3 means significant. B1 = 35 days prepartum; B2 = 7 days prepartum; B3 = 0 h postpartum. * *p* < 0.05, ** *p* < 0.01 and *** *p* < 0.001.

Rectal microorganisms were analyzed at the phylum and family levels. At the phylum level, *Firmicutes* and *Bacteroidetes* were the dominant species in the rectal flora, making up more than 98% of all species (Figure 4A). The relative abundance was 62.20% and 27.23% in group B1, 65.30% and 24.32% in group B2, and 66.61% and 22.59% in group B3, respectively. At the family level, dominant families were *Lachnospiraceae*, *Oscillospiraceae*, *Prevotellaceae*, and *Rikenellaceae*. Within the B1 group, relative abundance was 22.54%, 9.57%, 7.55%, and 7.32%; the B2 group was 22.56%, 9.60%, 8.86%, and 5.89%; the B3 group was 26.64%, 12.21%, 7.76%, and 6.52%, respectively (Figure 4B).

Changes in microbial communities at the phylum, family, and genus levels in the B1, B2, and B3 groups are shown in Figure 4C–E. At the phylum level, *Fibrobacterota* relative abundance was higher in group B1 than in groups B2 and B3 (Figure 4C). At the family level, the relative abundance of *Norank_f__p-251-o5*, *Lactobacillaceae*, *Fibrobacteraceae*, and *Moraxellaceae* was higher in group B1 than in group B2 and B3. The relative abundance of *Streptococcaceae* was higher in group B2 than in groups B1 and B3. However, the relative abundance of *Norank_f__F082* was higher in group B3 than in groups B1 and B2 (Figure 4D). At the genus level, the B1group had a higher relative abundance of *Norank_f__p-251-o5*, *Prevotella*, *Marvinbryantia*, and *Fibrobacter* relative abundance compared with groups B2 and B3. Group B2 had a higher relative abundance of *Streptococcus*, *Prevotellaceae_UCG-001*, *norank_f__Muribaculaceae* relative abundance compared with groups B1and B3. Group B3 had a higher relative abundance of *Norank_f__F082*, *Prevotellaceae_UCG-004*, and *Candidatus_Soleaferrea* relative abundance compared with groups B1 and B2 (Figure 4E).

Linear discriminant analysis (LDA) (Figure 4F) used for bacterial family discrimination found that the abundances of *Norank_f__p-251-o5*, *Prevotella*, *Marvinbryantia*, *Acinetobacter*, *Fibrobacter*, *Clostridium_sensu_stricto_3*, *hoa5-07d05_gut_group*, and *DEV114* were significantly higher in group B1 compared to the abundances in groups B2 and B3. The abundances of *Streptococcus*, *Prevotellaceae_UCG-001*, *norank_f__Muribaculaceae*, and *Acetitomaculum* were significantly higher in group B2 than those for groups B1 and B3. The abundances of *Prevotellaceae_UCG-004*, *Norank_f__F082*, *Lachnospiraceae_UCG-009*, *TM7a*, *Paracoccus*, *Leucobacter*, *Candidatus_Soleaferrea*, and *Brevundimonas* were significantly higher in group B3 than those for groups B1 and B2.

### 3.4. Metabolomic Profiles in the Serum and Identification of Metabolites

**Figure 5:**
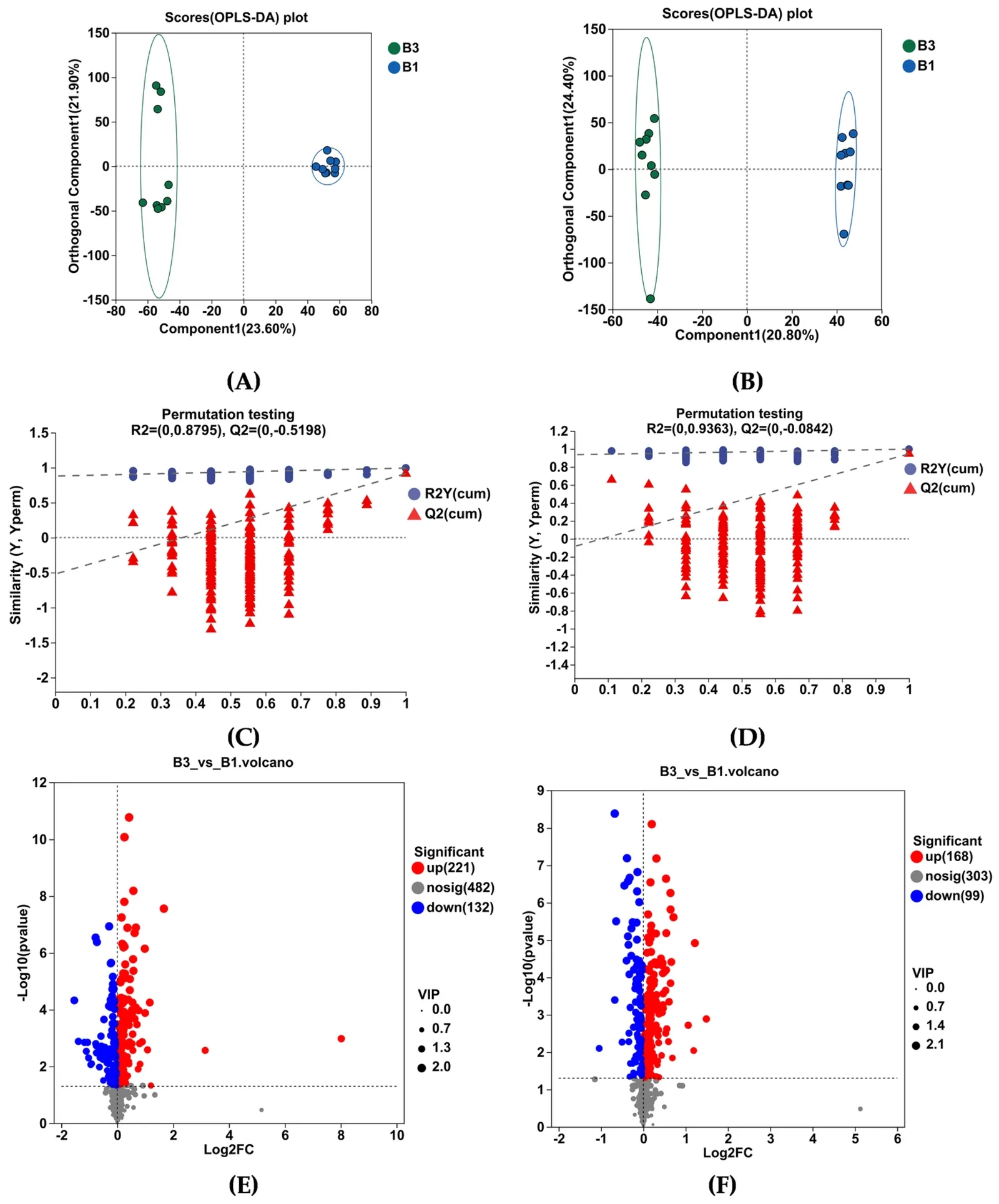

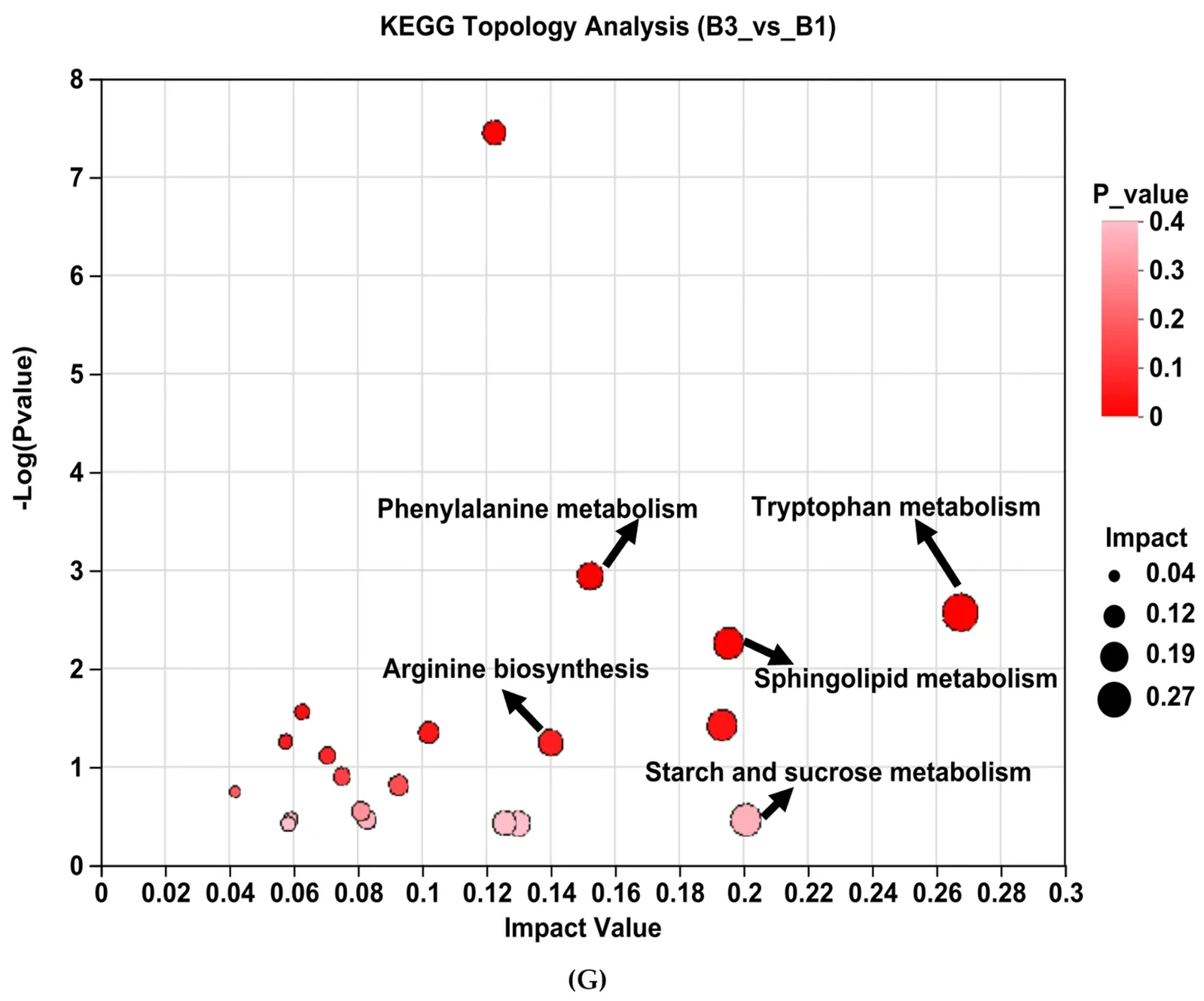
Serum metabolome profiles of group B3 and B1 Jennies. (**A**,**B**) show orthogonal partial least squares discrimination analysis (OPLS-DA) following positive ion electrospray ionization (ESI+) and negative ion electrospray ionization (ESI−) modes, respectively. (**C**,**D**) show OPLS-DA substitution test under ESI+ and ESI− modes, respectively. (**E**,**F**) show the volcano plots of serum differential metabolites under ESI+ and ESI− modes, respectively. (**G**) Metabolic pathway enrichment analysis of the differentially presented compounds between groups B3 and B1. B1 = 35 days prepartum, B3 = 0 h postpartum.

As shown by OPLS-DA in Figure 5A,B, the serum metabolome of the B1 and B3 groups was well separated. The software classification parameters were stable and correlated with fitness and prediction (Figure 5C,D). Both the positive and negative ion modes contain 18 samples and 6 quality control (QC) samples. Student’s *t*-test and fold difference analysis were performed. The selection of significantly different metabolites was determined based on the variable importance in the projection (VIP) obtained by the OPLS-DA model and the *p*-value of Student’s *t*-test, and the metabolites with VIP > 1, *p* < 0.05 were significantly different. A total of 620 differential metabolites were screened, among which 389 metabolites were upregulated, while 231 were downregulated (Figure 5E,F).

Analysis of KEGG pathway enrichment and the metabolites associated with metabolic pathways between groups B3 vs. B1 (Table 3). Enrichment pathways include tryptophan metabolism, phenylalanine metabolism, galactose metabolism, arginine biosynthesis, steroid hormone biosynthesis, glycerophospholipid metabolism, tyrosine metabolism, linoleic acid metabolism, sphingolipid metabolism, and starch and sucrose metabolism (Table 3 and Figure 5G).

### 3.5. Correlation Analysis Between Serum Metabolites and Serum Antioxidant Indicators, Inflammatory Indicators, Serum Biochemical Indicators, and Rectal Bacteria

**Figure 6:**
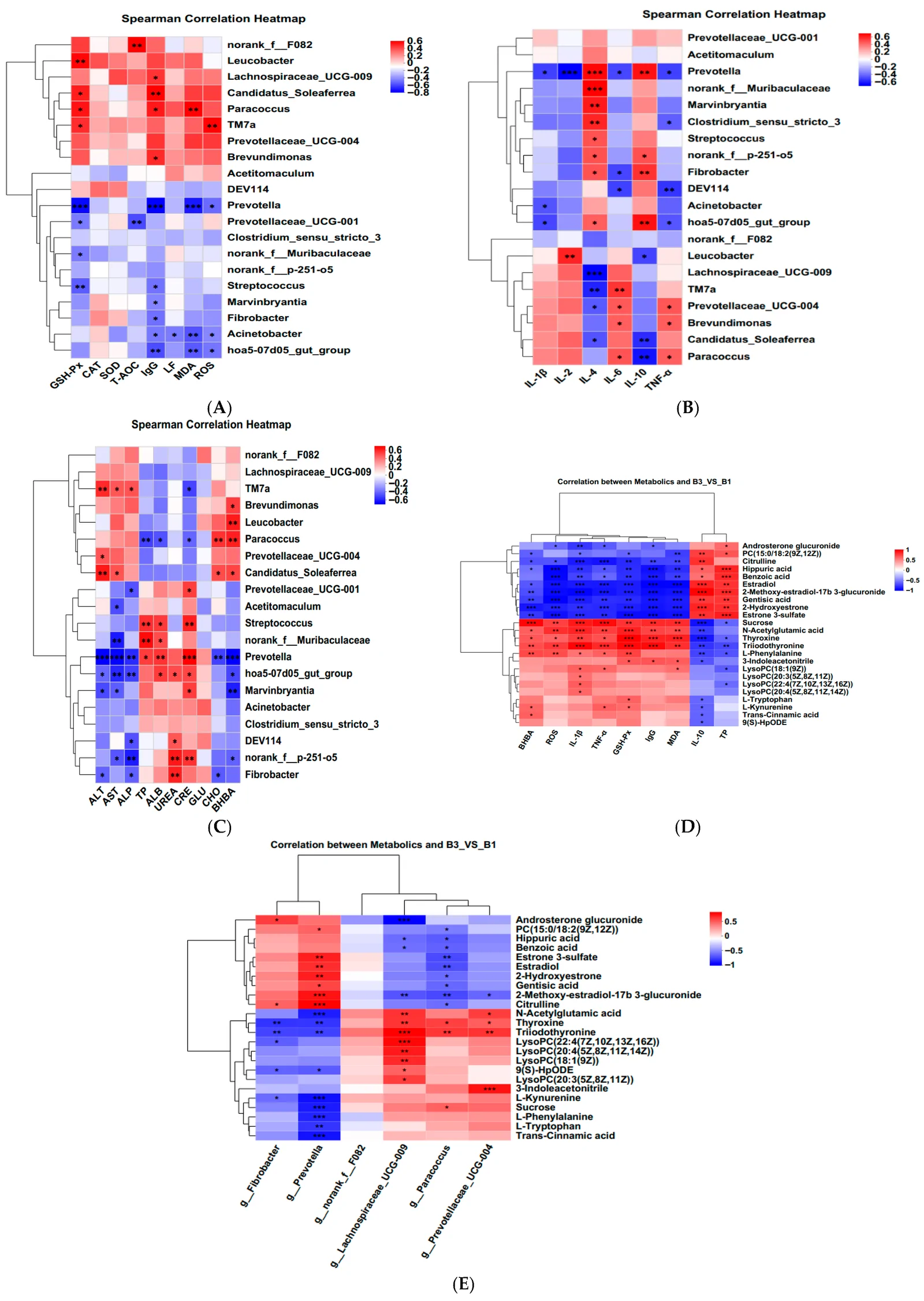
Spearman’s correlation analysis between rectal feces microbiota at the genus level by LEfSe analysis and (**A**). serum oxidative stress. (**B**) Inflammatory status. (**C**) biochemical parameters. (**D**) Correlation between metabolites and serum antioxidant indices, inflammatory status, and biochemical parameters. (**E**) correlation heat maps between serum metabolites and rectal microbiota. B1 = 35 days prepartum, B3 = 0 h postpartum. Blue color: negative correlation; Red color: positive correlation. * *p* < 0.05; ** *p* < 0.01; *** *p* < 0.001.

A correlation heatmap was constructed using Spearman’s correlation coefficient to examine the relationship between serum antioxidant status, immune cytokines, biochemical parameters, rectally differentiated bacteria, and serum metabolites (Figure 6A–E). The *p*-values and correlation coefficients for the associations between serum metabolites and serum antioxidant indicators, inflammatory indicators, serum biochemical indicators, and rectal bacteria are presented in Supplementary Tables S1 and S2.

## 4. Discussion

Female animals undergo physiological changes from late gestation to parturition, which results in increased oxidative stress and an inflammatory response [2,3]. Oxidative stress is an essential cause of enhanced inflammatory response and immunosuppression [18]. MDA is a marker of oxidative stress, and elevated levels indicate that the animal is under intense oxidative stress [19]. The concentrations of IL-1β, IL-2, IL-6, and TNF-α are organismal proinflammatory factors that play an essential role in the early inflammatory response. Delivery-related oxidative stress may further trigger immune and inflammatory abnormalities, increasing the risk of metabolic and infectious diseases [20,21]. Yang et al. [5] reported in their study of dairy cows that plasma TNF-α concentrations increased significantly from 7 days prepartum to the day of calving. The present study indicated that compared with group B3, the concentrations of MDA and IL-2 in the serum of Jennies in groups B1 and B2 were significantly decreased, while IL-10 showed the opposite result. In addition, compared with group B1, the concentrations of ROS, IL-1β, IL-6, and TNF-α were significantly increased in groups B2 and B3, whereas the concentration of IL-4 and IL-10 was significantly decreased. These results indicated that the oxidative stress level and inflammatory response of Jennies were gradually strengthened from 35 days before giving birth to the actual delivery. Jennies experienced higher oxidative stress and inflammatory states at 0 h postpartum, while enhanced antioxidant capacity corresponds to increased oxidative damage, the increases in the activities of GSH-Px and SOD, and the concentrations of IgG and LF supported the statement. Moderate oxidative stress can activate the body’s antioxidant defense mechanisms [22]. SOD catalyzes the degradation of superoxide radicals to oxygen and hydrogen peroxide, which CAT and T-AOC then degrade, which is an essential comprehensive index reflecting the total antioxidant capacity of an animal [23]. GSH-Px plays a key role in cellular resistance to oxidative stress [24]. Li et al. [25] reported that CAT activity and MDA content in serum significantly increased from 21 days prepartum to 1 day postpartum in female donkeys. Radin et al. [26] also observed similar trends in their study of peripartum goats. He et al. [27] proposed in their research of peripartum dairy goats that sustained high levels of GSH-Px and CAT before and after parturition indicate that the body has a strong ability to clear fat oxidation products.

It has been reported that there was immunosuppression in cows with a negative energy balance and that this immunosuppression may result from increased levels of relevant metabolites in the blood, such as BHBA and NEFA [28]. And β-oxidation of NEFA generates large amounts of ROS, and its excessive production may lead to increased oxidative stress [29]. In the present study, the concentrations of serum GLU were significantly higher in group B1 than in groups B2 and B3. In comparison, the concentrations of BHBA were significantly higher in group B3 than in the other two groups. In addition, the concentrations of serum ROS were significantly higher in groups B2 and B3 than in group B1. This indicates that groups B2 and B3 are in a state of metabolic stress, reflecting changes in energy metabolism, increased fat mobilization, and potential oxidative stress. At the same time, the decrease in serum GSH-Px activity in groups B1 and B2 also confirms this.

The intestinal flora and its metabolites play a crucial role as significant environmental regulators in modulating host metabolism and maintaining gut homeostasis [30], impacting animal health and various disease states. *Prevotella* belongs to the *Prevotellaceae* family, was confirmed as a key microbe that plays a significant role in enhancing disease prognosis [31]. *Fibrobacter* belongs to the *Fibrobacteres* phylum. *Fibrobacteria* are the main phylum responsible for lignin degradation and are also closely related to animal antioxidant levels. Guo et al. [14] reported that *Fibrobacter* showed a positive correlation with serum CAT and T-SOD activity in female donkeys, and a negative correlation with IL-2 concentration. In the present study, at the genus level, group B1 significantly upregulated the relative abundance of *Prevotella* and *Fibrobacter*, which were positively correlated with IL-4 and IL-10 concentrations and negatively correlated with IgG and IL-6 concentrations, indicating that compared with groups B2 and B3, group B1 of Jennies exhibited better anti-inflammatory function. The reasons were closely related to the increased relative abundance of the microbiota above.

*Acetitomaculum* is an aerobic bacterium with strong oxidizing ability and belongs to the Proteobacteria phylum. Koren et al. [9] reported that the maternal gut microbiota undergoes disruption in late gestation, characterized by a significant increase in the *Proteobacteria* and *Actinobacteria* phyla. The increase in *Proteobacteria* is a potential diagnostic microbial feature of gut microbiota dysbiosis and epithelial dysfunction [32,33]. The present study also found that the relative abundance of *Acetitomaculum*, *Prevotellaceae_UCG_001*, and *Streptococcus* was significantly increased in group B2, suggesting Jennies experienced a higher inflammatory response, and the results of the correlations between these different bacterial genera and inflammatory factors, as well as antioxidant parameters, also confirmed this point. Wang et al. [34] also found that the abundance of *Prevotellaceae* in the intestines of rats subjected to mild stress was significantly increased. Li et al. [35] reported that the relative abundance of *Streptococcus* in Jennies was positively correlated with the levels of systemic low-grade inflammatory biomarkers.

*Norank_f__F082* belongs to the *Bacteroidetes* phylum and is primarily involved in the degradation of non-cellulose. Li et al. [36] found that *norank_f__F082* was positively correlated with GSH-Px activity in goat rumen. *Lachnospiraceae* belong to the core of gut microbiota, participating in the production of metabolites such as short-chain fatty acids (SCFAs) and being closely associated with health [37]. A related study showed that the abundance of *Lachnospiraceae_UCG-009* was negatively correlated with elevated serum levels of inflammatory biomarkers in donkeys in early gestation [35]. In the present study, the relative abundance of *Norank_f__F082* and *Prevotellaceae_UCG-004* was significantly elevated in group B3. This shows that increased abundance of *Norank_f__F082* and *Lachnospiraceae_UCG-009* of may help the Jennies in group B3 reduce oxidative stress and damage, thereby enhancing their antioxidant activity.

Changes in serum metabolites can reflect antioxidant capacity and anti-inflammatory responses [16]. Research has shown that indole, a special microbial signal molecule in tryptophan metabolism, has a high concentration in the animal intestine and plays a wide range of roles in maintaining intestinal health and the body’s antibacterial and anti-inflammatory properties [38]. In addition, L-KYN is a potential endogenous antioxidant [39]. Arginine (Arg) promotes the production of immune proteins and the maturation of immune cells and significantly enhances humoral and cellular immunity. Studies have shown that Arg attenuates lipopolysaccharide-induced oxidative damage and apoptosis by promoting the expression of Nrf2 protein and increasing the levels of antioxidant enzymes (GPx1, CAT, and SOD2) [40]. Meanwhile, Hirakawa et al. [41] reported that N-acetylglutamic acid alleviated oxidative stress by regulating the expression of oxidative stress response genes in Arabidopsis thaliana and rice. Citrulline is a non-proteinogenic amino acid whose metabolism in the body involves not only the urea cycle but also arginine metabolism and nitric oxide synthesis [42]. In the present study, compared with group B1, group B3 upregulated L-tryptophan, L-kynurenine, and 3-indoleacetonitrile in tryptophan metabolism. At the same time, it also upregulated the level of N-acetylglutamic acid in the arginine metabolic pathway and downregulated the level of citrulline. Correlation analysis showed that L-Tryptophan and L-Kynurenine were significantly positively correlated with serum GSH-Px activity, while they were significantly negatively correlated with serum IL-10 concentration and *Prevotella* abundance. 3-Indoleacetonitrile showed a significant positive correlation with serum GSH-Px, IgG, MDA concentrations, and the abundance of the potential pathogenic bacterium *Prevotellaceae_UCG-004*, while showing a significant negative correlation with IL-10 concentration. N-Acetylglutamic acid was significantly positively correlated with serum GSH-Px, IgG, BHBA, ROS, IL-1β, TNF-α, MDA concentrations, and the relative abundance of *Lachnospiraceae_UCG-009* and *Prevotellaceae_UCG-004*. However, it was significantly negatively correlated with the relative abundance of *Prevotella*. Citrulline was significantly positively correlated with serum IL-10 concentrations and the relative abundance of *Prevotella*, while it was significantly negatively correlated with ROS, BHBA, IL-1β, TNF-α, MDA, and the relative abundance of *Paracoccus*. These results suggested that significantly enriched the tryptophan metabolism and arginine biosynthesis and their differential metabolites are the key pathways and metabolites causing enhanced oxidative stress and inflammatory responses of Jennies at parturition (Group B3), and the elevation or reduction in these differential metabolites may be associated with the proliferation of certain potential pathogenic bacteria, which could disrupt the balance of the rectal microbiota and thereby affect the host’s anti-inflammatory state. However, the above metabolic pathways and metabolites also enhanced antioxidant capacity, corresponding to increased oxidative damage.

Glycerophospholipid metabolism is closely related to choline production. Choline, Phosphatidylcholine, and Lysophosphatidylcholine all belong to the choline group of compounds and have a variety of physiological functions. LysoPC, a product of fatty acid oxidation, is produced when phosphatidylcholines (PC) are partially hydrolyzed by removing a fatty acid moiety [43] as well as inhibiting proinflammatory cell secretion [44]. As Chen et al. [45] demonstrated, oxidative stress may impair lipid metabolism and prevent the biosynthesis of cell membranes. PC (18:0/18:2 (9Z, 12Z)) acts as a ligand for apolipoprotein ApoA-I, promoting the formation of high-density lipoprotein and supporting cardiovascular health [46]. The present experiment showed that, compared with Group B1, the content of LysoPC (22:4(7Z,10Z,13Z,16Z)), LysoPC (20:3(5Z,8Z,11Z)), LysoPC (20:4(5Z,8Z, 11Z,14Z)), and LysoPC (18:1(9Z)) were significantly increased in group B3, while the content of PC (18:0/18:2 (9Z,12Z)) was significantly decreased. Among these, LysoPC (18:1 (9Z)) showed a significant positive correlation with serum IL-1β, TNF-α, and MDA levels, while exhibiting a significant negative correlation with TP concentration. PC (18:0/18:2 (9Z,12Z)) showed a significant negative correlation with serum BHBA, IL-1β, GSH-Px, and MDA levels, as well as the abundance of *Paracoccus*. It also showed a significant positive correlation with serum IL-10 and TP levels, as well as the abundance of *Prevotella*. These results suggest that these metabolites may be closely associated with enhanced lipid peroxidation processes, thereby improving the inflammatory response at parturition compared to late gestation (group B1). In addition, oxidative stress is associated with a variety of health problems, including intestinal diseases. Reports indicated that excessive sucrose intake promoted the progression of intestinal diseases in mice [47]. This study also found that, compared with group B1, group B3 showed an upregulation of sucrose levels in galactose metabolism. Correlation analysis revealed that sucrose was significantly positively correlated with serum BHBA, ROS, IL-1β, TNF-α, and MDA levels, as well as the abundance of *Paracoccus*, while it was significantly negatively correlated with IL-10 and TP levels, as well as the abundance of *Prevotella*. This suggests that enhanced oxidative stress during parturition (Group B3) is associated with elevated levels of sucrose in the galactose metabolism pathways.

**Figure 7:**
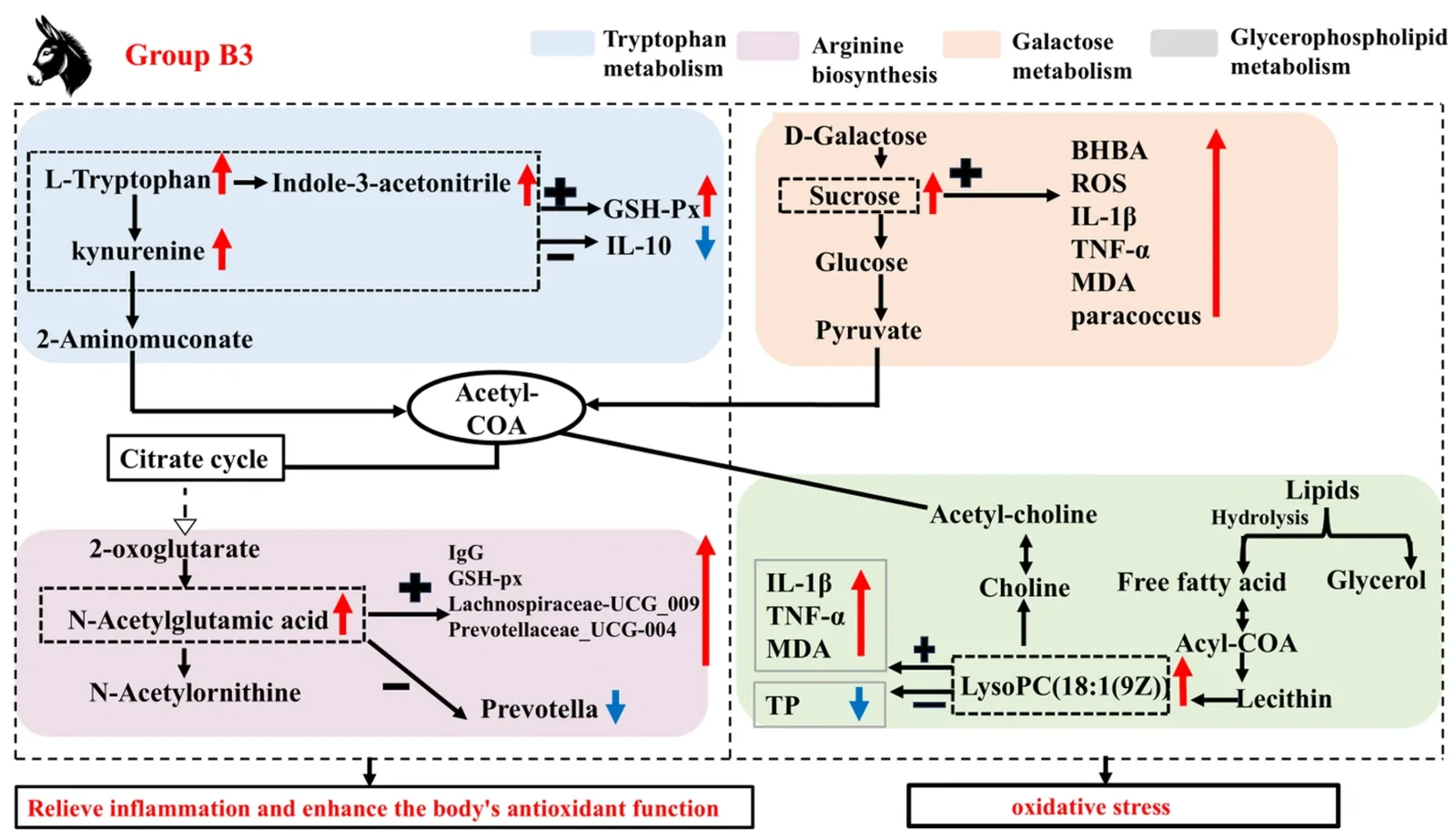
A mechanistic map of the microbiota and its involvement in enriched metabolic pathways. Note: GSH-Px (glutathione peroxidase); IgG (immunoglobulin G); IL-1β (interleukin-1β); TNF-α (tumor necrosis factor-α); ROS (reactive oxygen species); BHBA (β-hydroxybutyric acid); MDA (malondialdehyde); TP (total protein); Acyl-COA (Acyl-Coenzyme). “+” indicates a positive correlation between microorganisms and metabolites or other substances, and “−” indicates a negative correlation. “
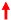
” represents a positive increase, “
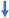
” represents a negative decrease.

In addition to these metabolic pathways, the present study revealed a significant enrichment of steroid hormone biosynthesis as an oxidized derivative of cholesterol, which is essential for maintaining the homeostasis of multiple physiological functions. This is similar to Zhang et al.’s study on donkeys [48]. Related reports indicate that estrogen also has important impacts on the immune system [49]. Specifically, estrogen can significantly reduce the production of IL-10 and TNF-α mediated by estrogen receptor α or estrogen receptor β in macrophages, while also regulating the NF-κB, AP-1, JNK, or ERK signaling pathways in human M1 and M2 macrophages [50]. These results indicate that the synthesis of steroid hormones and their conversion to estrogen play a key role in regulating the immune system. The current study found that, compared with group B3, the levels of Androsterone glucuronide, 2-Hydroxyestrone, Estrone 3-sulfate, Estradiol, and 2-Methoxy-estradiol-17β 3-glucuronide were significantly increased in group B1. Correlation analysis revealed that Androsterone glucuronide was significantly negatively correlated with serum ROS, IL-β, and TNF-α levels, while it was significantly positively correlated with TP levels and the relative abundance of *Fibrobacter*. It is suggested that the B1 group Jennies may enhance the serum anti-inflammatory response in late pregnancy by increasing the content of steroid hormone biosynthesis metabolic pathways and estrogen in the serum. This mechanism may help maintain the immune balance of the Jennies and promote the healthy development of the foal. Based on these analyses, Figure 7 shows the mechanism of influence of group B3 on the microbial community and metabolites. Similarly, Li et al. [16] demonstrated that host metabolism is associated with changes in microbial community abundance and composition, though the underlying mechanisms remain unclear.

In summary, Jennies experienced gradually elevated oxidative stress and inflammatory states from the 35 days prepartum to the parturition, with the inflammatory response being the most severe at parturition, and with enhanced antioxidant capacity corresponding to increased oxidative damage. Based on this finding, specific structural alterations in the rectal microbiota may modulate metabolic pathways of metabolites, thereby influencing the anti-inflammatory response in Jennies. Additionally, this study provides new insights into the potential mechanisms by which disruption of the “microbiome–metabolome” axis occurs during late gestation to parturition.

## 5. Conclusions

From 35 days prepartum to parturition, Jennies experienced a gradually intensifying oxidative stress and inflammatory states, with the inflammatory response being the most severe at parturition, and with enhanced antioxidant capacity corresponding to increased oxidative damage. Changes in the composition of the intestinal microbiota and metabolites may lead to this situation. Additionally, Spearman correlation analysis revealed a high degree of correlation between different bacterial genera and metabolite changes, suggesting that their combined effects may be the cause of enhanced antioxidant capacity and inflammatory status.

## Figures and Tables

**Table 1:** Experimental diet composition and nutritional level (dry-matter basis, %).

Item	Content
Ingredients	
Millet straw	44.63
Alfalfa hay	20.64
Corn	17.95
Soybean meal	8.27
Corn gluten meal	2.14
Corn germ meal	1.78
Wheat bran	1.71
Distiller’s fried grains with solubles	0.53
Extruded full-fat soybean	0.00
Premix ^1^	0.63
NaCl	0.46
CaCO_3_	0.41
CaHPO_4_	0.83
Total	100.00
Nutrient level, %	
Digestible energy, MJ/Kg ^2^	12.40
Crude protein	14.01
Ether extract	2.82
Neutral detergent fiber	52.48
Acid detergent fiber	31.15
Calcium	0.98
Phosphorous	0.31

^1^ One kilogram of premix provided the following: Vitamin A, 1,200,000 IU; Vitamin D, 250,000 IU; Vitamin E, 3000 IU; Fe, 4 g; Cu, 1.6 g; Zn, 12 g; Mn, 12 g; I, 72 mg; Se, 60 mg; Co, 100 mg. ^2^ Digestible energy was calculated according to the NRC (2007) [17].

**Table 2:** Serum biochemical indexes in late gestation and parturition.

Item	B1 ^1^	B2 ^1^	B3 ^1^	SEM ^2^	*p*-Value
ALT (U/L)	5.72 ^a^	8.72 ^ab^	13.64 ^a^	2.262	0.062
AST (U/L)	240.49 ^b^	268.42 ^b^	346.00 ^a^	14.164	<0.001
ALP (U/L)	83.67 ^b^	80.50 ^b^	116.77 ^a^	4.497	<0.001
TP (g/L)	70.36 ^a^	67.96 ^ab^	62.68 ^b^	1.885	0.025
ALB (g/L)	35.02 ^a^	33.54 ^ab^	31.07 ^b^	0.977	0.028
UREA (mmol/L)	8.98 ^a^	8.31 ^ab^	8.16 ^b^	0.263	0.085
CRE (μmol/L)	63.44 ^a^	62.94 ^a^	22.49 ^b^	2.064	<0.001
GLU (mmol/L)	4.48 ^a^	3.26 ^b^	3.76 ^b^	0.191	0.001
CHO (mmol/L)	1.72 ^b^	1.79 ^ab^	1.91 ^a^	0.044	0.018
BHBA (mmol/L)	0.20 ^b^	0.21 ^b^	0.27 ^a^	0.015	0.015

Superscript letters (a,b) within the same row indicate significant differences between experimental groups (*p* < 0.05). ^1^ B1 = 35 days prepartum; B2 = 7 days prepartum; B3 = 0 h postpartum. ^2^ SEM = standard error of the mean. Abbreviations: ALT = alanine aminotransferase, AST = aspartate aminotransferase, ALP = alkaline phosphatase, TP = total protein, ALB = albumin, UREA = urea, CRE = creatinine, GLU = glucose, CHO = cholesterol, BHBA = β-hydroxybutyric acid.

**Table 3:** Analysis of KEGG pathway enrichment and the metabolites associated with metabolic pathways between groups B3 vs. B1 ^1^.

Metabolic Pathways	KEGG ID	Hits ^2^	*p*_Value ^3^	Impact_Value	Upregulated	Downregulated
Tryptophan metabolism	map00380	6	0.003	0.267	L-Tryptophan	
					3-(2-(methylamino) ethyl)-1H-indol-5-ol	
					L-Kynurenine	
					3-Indoleacetic Acid	
					3-Indoleacetonitrile	
					Kynurenine	
Phenylalanine metabolism	map00360	5	0.001	0.152	Trans-Cinnamic acid	Phenylacetylglycine
					L-Phenylalanine	Hippuric acid
						Benzoic acid
Galactose metabolism	map00052	2	0.092	0.151	Sucrose	Dulcitol
Arginine biosynthesis	map00220	2	0.056	0.140	N-Acetylglutamic acid	Citrulline
Steroid hormone biosynthesis	map00140	12	0.000	0.122	18-Hydroxycorticosterone	Dehydroepiandrosterone
					Cortisol	5alpha-Pregnan-20alpha-ol-3-one
						Estrone sulfate
						Androsterone glucuronide
						Testosterone glucuronide
						2-Hydroxyestrone
						Estrone 3-sulfate
						Estradiol
						Estrone 3-glucuronide
						2-Methoxy-estradiol-17b 3-glucuronide
Glycerophospholipid metabolism	map00564	10	0.044	0.102	LysoPC (22:4(7Z, 10Z,13Z,16Z))	PS (18:0/20:4(8Z,11Z,14Z,17Z))
					LysoPC (20:3(5Z,8Z,11Z))	LysoPC (15:0)
					LysoPC (20:4(5Z,8Z,11Z,14Z))	PC (15:0/18:2(9Z,12Z))
					LysoPC (18:1(9Z))	LPC (18:3)
					LysoPC (20:3(8Z, 11Z,14Z))	
					LysoPC (16:1(9Z)/0:0)	
Tyrosine metabolism	map00350	6	0.001	0.007	Thyroxine	Dopaquinone
					Phenol	Gentisic acid
					Metanephrine	
					Triiodothyronine	
Linoleic acid metabolism	map00591	3	0.001	0.000	9(S)-HpODE	PC (15:0/18:2(9Z,12Z))
					13(S)-HpODE	

^1^ (B1 = 35 days prepartum, B3 = 0 h postpartum); ^2^ Hits represent the number of KEGG compound IDs annotated to pathways in this metabolism; ^3^ The *p*-value is uncorrected, and *p*-values less than 0.05 are considered enrichment terms.

## Data Availability

The serum metabolomics data have been deposited to MetaboLights repository with the study identifier MTBLS12897 at https://www.ebi.ac.uk/metabolights/MTBLS12897 (accessed on 26 August 2025), while the microbiome diversity data can be found in the NCBI Sequence Read Archive (SRA) under accession numbers PRJNA1293311.

## Supplementary Materials

The following supporting information can be downloaded at: https://www.mdpi.com/article/10.3390/antiox14101253/s1, Table S1. Correlation analysis of rectal bacteria with serum antioxidant and inflammatory indicators and serum biochemical indexes. Table S2. Correlation analysis between serum metabolites and serum antioxidant indicators, inflammatory indicators, serum biochemical indicators, and rectal bacteria (Partial).
