# Supplementary material for: Rectal Microbiomes and Serum Metabolomics Reveal Changes in Serum Antioxidant Status and Immune Responses of Dezhou Donkeys in Late Gestation to Parturition
Source: Antioxidants (Basel). 2025 Oct 18;14(10):1253. doi: 10.3390/antiox14101253 (PMC12561646; doi:10.3390/antiox14101253)
Supplement: Supplementary file 1 [file antioxidants-14-01253-s001.zip › antioxidants-3905237-supplementary.pdf]

Supplementary Table S1. Correlation analysis of rectal bacteria with serum antioxidant and inflammatory indicators and serum biochemical indexes.

Rectal bacterial	T-AOC		GSH-Px		IgG		IL-1β		IL-2		IL-4		IL-6		IL-10		TNF-α		MDA		BHBA		ALT		AST		ALP		CHO		CRE	
	R	p	R	p	R	p	R	p	R	p	R	p	R	p	R	p	R	p	R	p	R	p	R	p	R	p	R	p	R	p	R	p
Norank_f_F082	0.53	**	—	—	—	—	—	—	—	—	—	—	—	—	—	—	—	—	—	—	—	—	—	—	—	—	—	—	—	—	—	—
Lachnospiraceae_UCG-009	—	—	—	—	0.40	*	—	—	—	—	-0.63	***	—	—	—	—	—	—	—	—	—	—	—	—	—	—	—	—	—	—	—	—
Prevotella	—	—	-0.71	***	-0.75	***	-0.47	*	-0.64	***	0.61	***	-0.45	*	0.58	**	-0.47	*	-0.67	***	-0.66	***	-0.65	**	-0.63	***	-0.55	**	-0.49	**	0.63	***
Fibrobacter	—	—	—	—	-0.47	*	—	—	—	—	0.43	*	-0.5	*	0.51	*	—	—	—	—	—	—	—	—	—	—	—	—	—	—	—	—
Prevotellaceae_UCG-001	-0.56	**	-0.45	*	—	—	—	—	—	—	—	—	—	—	—	—	—	—	—	—	—	—	—	—	—	—	-0.45	*	—	—	0.46	*
Prevotellaceae_UCG-004	—	—	—	—	—	—	—	—	—	—	-0.42	*	0.42	*	—	—	0.42	*	—	—	—	—	0.41	*	—	—	—	—	—	—	—	—

Note: R denotes the correlation coefficient, p denotes the p-value, and "—" indicates no significant difference. * *p*< 0.05; ** *p*< 0.01; *** *p*< 0.001.

Supplementary Table S2. Correlation analysis between serum metabolites and serum antioxidant indicators, inflammatory indicators, serum biochemical indicators, and rectal bacteria (Partial).

Item	GSH-Px		IgG		IL-1β		IL-10		TNF-α		MDA		BHBA		ROS		Lachnospiraceae_UCG-009		Prevotellaceae_UCG-004		Prevotella		Paracoccus	
	R	p	R	p	R	p	R	p	R	p	R	p	R	p	R	p	R	p	R	p	R	p	R	p
N-acetylglutamic acid	0.74	***	0.62	**	0.73	***	-0.65	**	0.65	**	0.60	**	0.54	*	0.69	**	0.62	**	0.59	*	-0.77	***	—	—
citrulline	-0.70	**	-0.62	**	-0.75	***	0.69	**	-0.77	***	-0.62	**	-0.58	*	-0.57	*	—	—	—	—	0.77	***	-0.52	*

Note: R denotes the correlation coefficient, p denotes the p-value, and "—" indicates no significant difference. * *p*< 0.05; ** *p*< 0.01; *** *p*< 0.001.
